# From cyanochemicals to cyanofactories: a review and perspective

**DOI:** 10.1186/s12934-015-0405-3

**Published:** 2016-01-08

**Authors:** Jie Zhou, Taicheng Zhu, Zhen Cai, Yin Li

**Affiliations:** CAS Key Laboratory of Microbial Physiological and Metabolic Engineering, Institute of Microbiology, Chinese Academy of Sciences, No 1, West Beichen Road, Chaoyang District, 100101 Beijing, China

**Keywords:** Cyanobacteria, Cyanochemicals, Cyanofactories, Rubisco, Metabolic Engineering, Rerouting carbon flux, Strong promoter, Cofactor balance

## Abstract

Engineering cyanobacteria for production of chemicals from solar energy, CO_2_ and water is a potential approach to address global energy and environment issues such as greenhouse effect. To date, more than 20 chemicals have been synthesized by engineered cyanobacteria using CO_2_ as raw materials, and these studies have been well reviewed. However, unlike heterotrophic microorganisms, the low CO_2_ fixation rate makes it a long way to go from cyanochemicals to cyanofactories. Here we review recent progresses on improvement of carbon fixation and redistribution of intercellular carbon flux, and discuss the challenges for developing cyanofactories in the future.

## Background

Cyanobacteria are photoautotrophic prokaryotes, which can directly convert CO_2_ into organic compounds using solar energy via photosynthesis. They are important primary producers and it is estimated that 20–30 % organic carbon on the earth is derived from photosynthetic carbon fixation by cyanobacteria [1]. Even though cyanobacteria and higher plants are performing oxygenic photosynthesis, the photosynthetic efficiency of cyanobacteria is tenfold higher than that of higher plants [2]. Moreover, the growth cycle of cyanobacteria is much shorter than that of higher plants: a recent study showed that a cyanobacterial strain can complete one generation cycle within approximately 2 h [3]. In addition, the gene manipulation of cyanobacteria is much easier than that of higher plants and eukaryotic photosynthetic algae. Therefore, engineering cyanobacteria into cyanofactories is an attractive approach to use solar energy and recycle CO_2_ and hence address global energy and environmental issues.

**Fig. 1:**
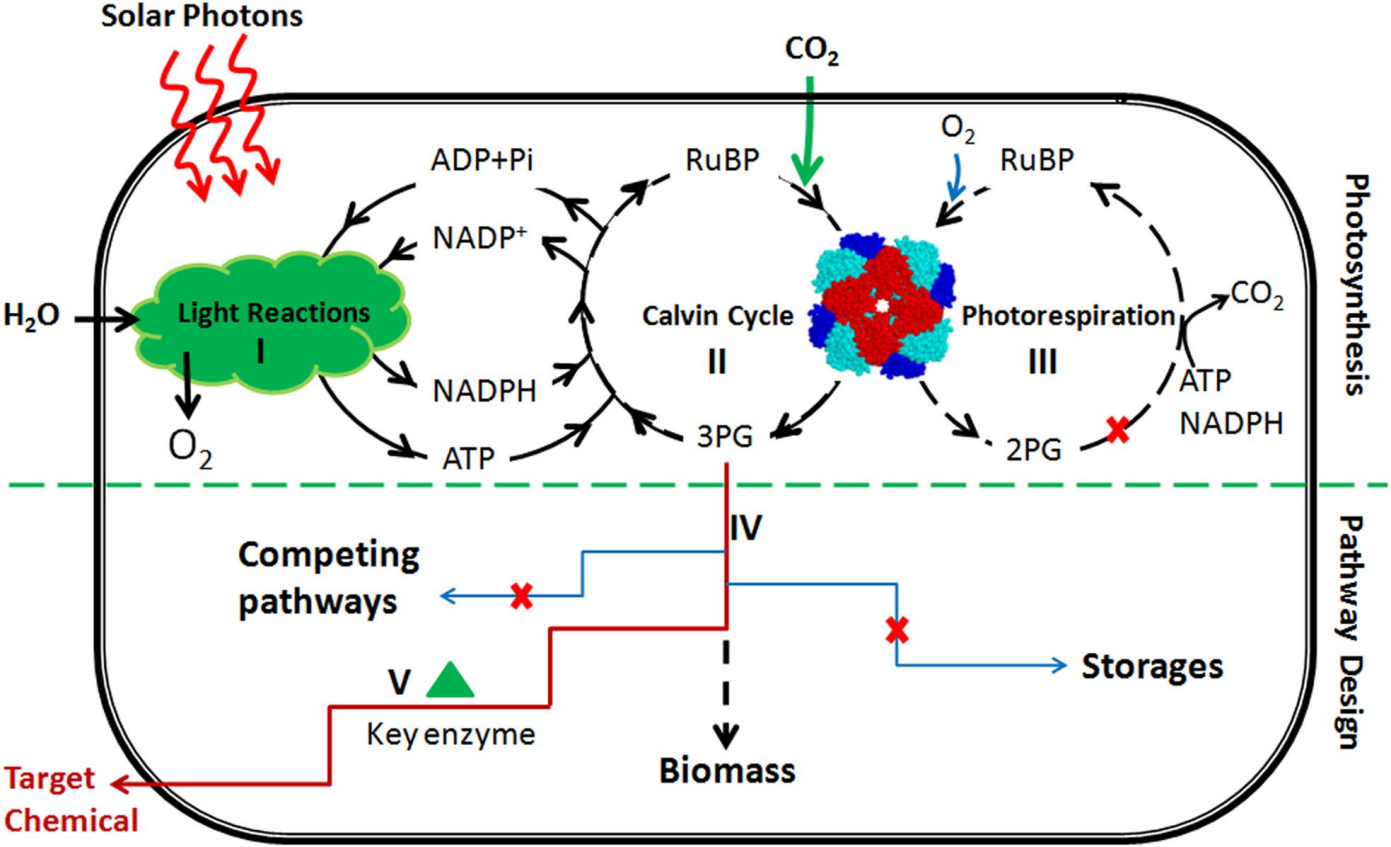
Strategies of genetic engineering for improvement of chemicals production from CO_2_ in cyanobacteria. Improvement of photosynthesis including enhancing light reactions (*I*), optimizing Rubisco to increase the efficiency of Calvin cycle (*II*) and disrupting photorespiration pathway to reduce photorespiration (*III*). Target pathway designs include blocking competing pathways and storages pathways (*IV*), and increasing expression level of key enzymes to drive more carbon flux towards target pathways, with the consideration of co-factor balance by using NADPH-dependent enzymes (*V*)

In the past 15 years, more than 20 chemicals have been synthesized from CO_2_ by cyanobacteria (hereafter referred as cyanochemicals). These include C2 chemicals, such as ethanol [4] and ethylene [5]; C3 chemicals, such as acetone [6] and isopropanol [7]; C4 chemicals, such as butanol [8] and 2,3-butanediol [9]; and C5 chemical isoprene [10, 11]. These cyanochemicals have been well reviewed [11, 12]. Theoretically, most of the chemicals that can be produced from sugar through heterotrophic microorganisms can also be produced from CO_2_ by engineered cyanobacteria. However, the titer and productivity of cyanochemicals are much lower than that expected. Most cyanochemicals were produced at levels of mg/L, except few chemicals that were produced in g/L (Table 1): i.e., isobutyraldehyde (1.10 g/L) [8], d-lactate (1.06 g/L, 1.14 g/L) [13, 14], 2,3-butanediol (2.38 g/L) [9], sucrose (3.50 g/L) [15] and ethanol (5.50 g/L) [4]. Generally the productivity of cyanochemicals is between 0.2 µg–46 mg/g dry cell weight (DCW)/h [16], which is at least 100-fold lower than production of ethanol from glucose. Improving cyanochemicals production, with the consideration of improving CO_2_ fixation efficiency, distribution of endogenous carbon flux, redox balance and product conversion efficiency (Fig. 1), will facilitate the development of cyanofactories.

**Table 1:** Production of bulk chemicals from CO_2_ in cyanobacteria at g/L scale

Host	Chemical	Titer (g/L)	Strategies toward increased production
7942	Isobutyraldehyde [8]	1.10	Overexpression of *rbcLS* genes; Supplement of 50 mM NaHCO_3_
6803	d-Lactate [14]	1.06	Introducing a more efficient enzyme DldhL with a super-strong promoter; blocking poly-3-hydroxybutyrate and acetate pathways from acetyl-CoA node
6803	d-Lactate [15]	1.14	Introducing a more efficient enzyme GlyDH; expression of a soluble transhydrogenase
7942	2,3-Butanediol [9]	2.38	Selection of key enzyme sADH with higher efficiency; using NADPH-dependent sADH; supplement of 50 mM NaHCO_3_
7942	Sucrose [15]	3.50	Expressing a symporter of protons and sucrose; minimizing glucose or sucrose consuming reactions
6803	Ethanol [4]	5.50	Selection of key enzymes with higher activity; blocking poly-3-hydroxybutyrate synthetic pathway

## Improving CO_2_ fixation in cyanobacteria

Usually, sufficient supply of substrate (e.g. glucose) for heterotrophic microorganisms is not a problem. The situation is more complicated when CO_2_ is used as the sole substrate, as the CO_2_ concentration in aqueous solution is low and CO_2_ fixation is thus an inefficient process. For example, isobutyraldehyde production was improved by overexpression of ribulose-1,5-bisphosphate carboxylase/oxygenase (Rubisco), the key enzyme of Calvin cycle, in cyanobacterium *Synechococcus elongatus* PCC 7942 (*S*. 7942) [8], and the titer of d-lactate produced by *Synechocystis* sp. PCC 6803 (*S*. 6803) was increased by addition of acetate as an extra organic carbon source [14], indicating that the supply of endogenous organic carbon is not sufficient for production of chemicals in cyanobacteria. Therefore, increasing the efficiency of photosynthetic CO_2_ fixation can improve production of cyanochemicals.

### Molecular engineering of Rubisco

Rubisco catalyzes carboxylation reaction of ribulose-1,5-biphosphate (RuBP) with CO_2_ and initiates the Calvin cycle in photosynthetic organisms. However, the extremely low carboxylation efficiency of Rubisco and its competing oxygenase activity has made this enzyme a rate-limiting one during the photosynthetic CO_2_-fixation process. Tremendous efforts have been made to engineer Rubisco to increase its carboxylation activity as well as to decrease its oxygenation activity, but limited success has been achieved [17–19] due to its complex structure–function relationships and lack of an efficient selection system towards its activity.

The selection system using phosphoribulokinase (PRK)-expressing *Escherichia coli* (*E. coli*) has been reported for directed evolution of Rubisco [20–22]. The selection rationale for this system is “PRK poisoning followed by Rubisco rescuing”: the catalytic product of PRK, RuBp, is a dead-end product and causes growth arrest of *E. coli*. Rubisco mutants that efficiently convert ribulose-1,5-bisphosphate to glycerate-3-phosphate have been selected and cell growth was restored. However, this type of selection system appeared to improve the expression rather than activity. To address this question, we reported an improved selection system by saturating Rubisco’s functional expression in *E. coli* via optimization of the host/promoter combinations and overexpression of the specific Rubisco chaperon protein RbcX [23]. The evolution path for Rubisco towards improved expression was blocked by using an improved selection system and up to 85 % increment of specific carboxylation activity was achieved from a *Synechococcus* sp. PCC7002 (*S*. 7002) Rubisco mutant [23]. Sequence and structural analyses revealed that two single mutations in the small subunit (E49V^RbcS^, D82G^RbcS^) conferred the improved activity. The two mutations are far away from any conserved catalytic residues in the large subunit, this further emphasizing the contribution of the small subunit to the holoenzyme activity. In 2015, Durao et al. also engineered Rubisco using an improved selection system based on the saturated Rubisco functional expression in *E. coli* [24]. However, the authors used chaperon GroESL from *E. coli* instead of the RbcX from *Synechococcus* to facilitate Rubisco expression in *E. coli*, based on their finding that RbcX failed to assemble, perhaps due to that some Rubisco mutants contain mutations which affected the binding of RbcX–Rubisco. Using this selection system, a single mutation in the large subunit (F140I^RbcL^) of *S*. 6803 Rubisco significantly increased the carboxylation efficiency by 2.9-fold and slightly reduce the specificity by 9 % [24]. Moreover, upon introducing this mutant into *S*. 6803, photosynthesis rate was improved by approximately 55 % [24], which provides the first direct evidence that a single catalytic efficiency-improved Rubisco is capable of improving the photosynthesis rate of the host.

### Reinforcing cyanobacterial carbon concentrating mechanism

Rubisco has inherent flaws that includes extremely low carboxylation efficiency and competitive inhibition of O_2_. To avoid the competing oxygenase activity, the carboxylation reaction of Rubisco is confined in the carboxysome of cyanobacteria where the carbon concentrating mechanism (CCM) is working [25, 26]. CO_2_ is transported to cyanobacterial plasma membrane in the form of bicarbonate by bicarbonate transporter, then converted to CO_2_ by carbonic anhydrase (CA), and finally CO_2_ is carboxylated by Rubisco in the carboxysome [25]. Therefore, co-overexpression of Rubisco and CA in carboxysome to certain ratio may contribute to CO_2_ fixation in cyanobacteria.

Increasing the activity of bicarbonate transporter and/or CA will contribute to the improvement of Rubisco’s CO_2_ fixation rate. Three types of bicarbonate transporters and two types of CA have been characterized so far in cyanobacteria [26]. A recent study demonstrated that introducing an extra bicarbonate transporter resulted in a twofold increase of growth and biomass [27]. Furthermore, overexpression of CA was also shown to increase the heterotrophic CO_2_ fixation in *E. coli* [28]. These studies indicated that sufficient supply of inorganic carbon has crucial value for the enhancement of carbon fixation in cyanobacteria.

### Engineering photorespiration

Due to the complexity of oxygenic photosynthesis and oxygenase activity of Rubisco, it is difficult to improve the efficiency of photosynthetic carbon fixation. Being the biochemical process along with the Calvin cycle, photorespiration leads to approximately 25 % loss of the fixed carbon [29]. Reducing or blocking photorespiration, therefore, might be a good solution to increase the efficiency of photosynthetic carbon fixation. However, as the important physiological function of photorespiration is to protect photosynthetic organisms from photoinhibition, most attempts to increase photosynthesis efficiency by reducing photorespiration have been unsuccessful [30]. According to the study conducted in 2007, by introducing *E. coli* glycolate catabolic pathway to chloroplasts of *Arabidopsis thaliana* photorespiration was reduced and photosynthetic efficiency as well as biomass production was significantly improved [31].

Due to the CCM activity, it had been thought that photorespiration was not existing in cyanobacteria, until the discovery of photorespiratory metabolism in *S*. 6803 [26, 32]. Another CO_2_ fixation pathway based on 3-hydroxypropionate bicycle was introduced into cyanobacterium *S*. 7942 in order to re-fix the CO_2_ released from photorespiration [33]. However, no significant increase of growth and photosynthesis was observed.

## Rerouting endogenous carbon flux

Photosynthesis and CO_2_ fixation make the metabolism of cyanobacteria more complicated than that of heterotrophic microorganisms. Moreover, with the accessibility of all basic techniques [34, 35], the development of genetic manipulations in cyanobacteria lags far behind that of *E. coli*. To date, there are only few successful examples that have been reported for genetic manipulation of native carbon flux in cyanobacteria. Due to the limited carbon fixation capability and difficulty of improving photosynthetic efficiency, rerouting intracellular carbon flux becomes very important to increasing production of cyanochemicals. Here we mainly describe the useful strategies for rerouting endogenous carbon flux: blocking synthesis of endogenous storage carbohydrates, such as glycogen and poly-3-hydroxybutyrate (PHB), blocking competing pathways, and reinforcing native biosynthetic pathways.

### Blocking glycogen synthetic pathway

In cyanobacteria, the photosynthetically fixed carbon is usually used for biomass accumulation or stored as glycogen [36]. Impairing of this synthetic pathway of glycogen synthesis does not affect the growth of *S*. 6803 under continuous light condition [37], suggested that glycogen is not compulsory for cell growth. Production of pyruvic acid and 2-oxoglutaric acid was significantly increased in glycogen-deficient strains under nitrogen limited conditions [37]. This evidence suggests the production of pyruvic acid-dependent and 2-oxoglutaric acid-dependent chemicals can be improved by blocking glycogen synthetic pathway. A recent study also showed that lactate production rate was increased by twofold in glycogen-deficient *S*. 6803 strain under the nitrogen limited condition [38]. Furthermore, sucrose secretion was significantly increased by blocking glycogen synthesis or accelerating glycogen breakdown under salt stress conditions [15]. Thus rerouting carbon flux from glycogen synthesis is expected to be a useful strategy for developing cyanofactories.

### Blocking PHB synthetic pathway

In addition to glycogen, PHB is another storage carbon source in cyanobacteria. PHB can be accumulated up to 40 % of dry cell weight in *S*. 6803 under multiple stress conditions, such as nitrogen and phosphate limited conditions [39, 40]. Blocking PHB synthetic pathways to improve production of cyanochemicals was first demonstrated in acetone production in cyanobacteria. Acetone production by *S*. 6803 was increased from undetectable levels to detectable levels by blocking PHB synthetic pathway under stress conditions [6]. Subsequently, production of 3-hydroxybutyrate [41],d-lactate [13] and butanol [42] were also improved by blocking PHB synthetic pathway in *S*. 6803. Thus, multiple stresses are major contributing factors for the accumulation of PHB [39, 40]. Blocking PHB synthetic pathway did not increase l-lactate production in *S*. 6803 [38], which is perhaps because nitrogen was the only limited condition applied in that study.

Blocking the synthesis of storage compounds does not inhibit cell growth under normal growth conditions [13, 37], while multiple stress conditions such as dark, salt stress, nitrogen and phosphate limitations contribute to the accumulation of storage carbohydrates [15, 39, 40]. Therefore, blocking the synthesis of storage compounds is suitable for cyanochemicals production in a two-stage-process, in which biomass is accumulated under normal culture conditions, then target chemical production is initiated under stress conditions. The advantage of the two-stage-process is to avoid toxic effects of target chemicals on cells growth. The disadvantage is the low productivity of chemicals, as the total amount of chemicals that can be produced will not exceed the amount of biomass accumulated. Therefore, the two-stage-process might be suitable to produce high value products in cyanobacteria.

### Blocking competing pathways

Although this strategy has been widely used in metabolic engineering of heterotrophic microorganisms, initially this strategy was used in cyanobacteria for the production of sucrose, wherein the synthesis of glycogen was blocked in *S*. 6803 to increase the accumulation of sucrose [43]. Since then, the strategy of blocking competing pathway has been gradually used for production of cyanochemicals [34]. For example, acetone and d-lactate productions was increased by sixfold and twofold by blocking acetate synthetic pathway in *S*. 6803, respectively [6, 13]. Moreover, disruption of oxaloacetate synthesis from phosphoenolpyruvate can drive more carbon flux to lactate production [44].

#### Reinforcing native biosynthetic pathway

Rerouting the flux towards the synthesis of the direct substrate of the target chemical is another useful strategy to increase production of chemicals. For examples, improving synthesis of pyruvate via overexpression of pyruvate kinase resulted in a substantial increase of lactate production [44] and enhancing acetyl-CoA level via overexpression of phosphoketolase resulted in a significant increase of butanol titer [42]. Recently, improvement of tricarboxylic acid (TCA) cycle resulted in a 10 % increased carbon flux towards ethylene synthetic pathway in *Synechocystis* [5].

### Reinforcing the introduced biosynthetic pathways

#### Using strong promoters

When carbon flux is sufficient, the efficiency of the introduced synthetic pathway is crucial for chemicals production. However, genetic manipulation tools for cyanobacteria lag behind what has been developed for *E*. *coli,* and genetic manipulation tools developed in *E. coli* often do not function as designed in cyanobacteria [45]. Due to lack of strong promoters, increasing expression levels of key enzymes is currently a main task for increasing flux towards the target chemicals. *E. coli* strong promoters such as P_trc_ and P_lac_ are usually used for cyanochemicals production, e.g. butanol [46], ethylene [47] and lactate [44]. In the lactate study, different promoters with different strength were used to drive key enzyme lactate dehydrogenase expression and the data showed that stronger promoters can achieve higher lactate production [44]. Cyanobacterial native promoters such as P_rnpB_, P_cpc_, P_rbc_, and P_psbA2_ were also successfully used for cyanochemicals production, e.g. lactate [48], ethanol, acetone [6] and isoprene [10, 49]. Various promoters (P_rnpB_, P_psbA2_, and P_trc_) were analyzed regarding their ability to drive expression of L-lactate dehydrogenase (LDH) in *S*. 6803 and the expression level of LDH showed that further improvement are still required [48].

To increase the expression level of key enzymes involved in the introduced synthetic pathways in cyanobacteria, a strong promoter P_cpc560_ was identified [50]. Using P_cpc560_, the expression level of heterologous protein can account for as much as 15 % of total soluble proteins in *S*. 6803, a level comparable to *E. coli* [50]. d-lactate production was improved in *S*. 6803 using the super-strong promoter [13]. However, as the native super-strong promoter P_cpc560_ is involved in phycocyanin synthesis and is tightly regulated by light conditions in cyanobacteria [51], not all genes can be expressed to a high level using P_cpc560_. Further optimization of P_cpc560_ promoter and other strong promoters is therefore needed.

Fusing target genes with the endogenous *cpcB* gene encoding the phycocyanin β-subunit is an alternative approach to increase the expression of exogenous genes under the native *cpc* operon promoter [52]. Using this strategy, the expression level of the fusion protein reached up to 20 % of total cellular proteins and a 100-fold yield of β-phellandrene hydrocarbons was obtained [52].

#### Increasing copy number of target genes

Increasing copy number of target genes is another useful approach to increase expression level of key enzymes. For example, ethanol production was increased to 5.5 g/L from approximately 1 g/L via introduction of two copies of the *adc* gene into *S*. 6803 [4].

#### Using inducible promoters to control gene expression

For a synthetic pathway involving several enzymes, the key enzyme usually needs to be expressed to a high level, and the proportion of each enzyme is crucial to increase the efficiency of the synthetic pathway. A Previous study showed that the inducible promoters for *E. coli* did not work well in cyanobacteria [9]. Recently, an inducible promoter library based on *S*. 6803 *cpcB* promoter and a RBS library of *S*. 7002 were developed, which will contribute to controlling expression levels of enzymes in cyanobacteria [35].

## Improving the compatibility between the introduced pathways and cellular metabolism

Heterotrophic microorganisms are NADH-rich microbes and most their cellular enzymes are NADH-dependent. Remarkably, the photosynthetic prokaryote cyanobacteria are NADPH-rich microbes, as large amount of NADPH is generated in photosynthesis light reactions. Efficient utilization of abundantly available NADPH is important for improving the compatibility between the introduced pathways and the native cellular metabolism.

### Using NADPH-dependent enzymes

A NADPH-dependent alcohol dehydrogenase (YqhD) was compared with two NADH-dependent alcohol dehydrogenases for production of isobutanol in cyanobacterium *S*. 7942, and the YqhD was shown to be the most active in *S*. 7942 [8]. In addition, butanol production was increased by fourfold by replacing the NADH-dependent alcohol dehydrogenase with the NADPH-dependent alcohol dehydrogenase [53]. Moreover, 2,3-butanediol production was significantly improved by using the NADPH-dependent secondary alcohol dehydrogenase (sADH) to create a cofactor-balanced biosynthetic pathway [9].

### Converting NADPH to NADH

Because of the lack of natural NADPH-dependent enzymes, converting cellular NADPH to NADH is another useful approach to achieve cofactor balance in engineered cyanobacteria. One strategy is co-expression of transhydrogenase to accelerate the conversion of NADPH to NADH to provide enough NADH for NADH-dependent enzymes [14, 54]. Co-expression of transhydrogenase increased the production of lactate [14, 54]. Manipulations of the transhydrogenase expression level may be necessary to meet the specific cofactor demand. Shifting cofactor specificity of enzymes from NADH-dependent to NADPH-dependent via site-directed mutagenesis is another useful strategy. In previous efforts to increase production of l-lactate from CO_2_ in cyanobacteria, cofactor specificity of the NADH-dependent l-lactate dehydrogenase (LDH) was shifted to NADPH-dependent via site-directed mutagenesis to increase its activity on NADPH [44]. However, the activity of the engineered LDH on NADPH was much lower than that of the wild-type l-LDH using NADH as cofactor. In addition, the activity of the engineered LDH on NADH was also significantly decreased [44], indicating that an alternative strategy is required to shift cofactor specificity of enzymes from NADH-dependent to NADPH-dependent to improve cyanochemicals production.

## Future perspectives

Cyanofactories remain far less efficient than heterotrophic cell factories like those based on *E. coli* and *Saccharomyces cerevisiae* (*S. cerevisiae*). The specific glucose uptake rate of *E. coli* and *S. cerevisiae* can reach 900~2700 mg/gDCW/h [55], while the cyanobacterial CO_2_ fixation rate is within the range of 3.5~24.1 mg/gDCW/h [16]. Although more than 50 % of the fixed carbon has been successfully converted into cyanochemicals in the case of sucrose [15] and lactic acid [44], the yield of many chemical synthetic pathways reported to date is still very low. Exploitation of the following opportunities in metabolic engineering and synthetic biology will contribute to development of efficient cyanofactories.

### Gaining new insights into the cyanobacterial metabolism

Current knowledge on cyanobacteria is far from being complete. The functions of many genes need to be assigned, and the metabolisms and their regulations are yet to be elucidated. For example, the TCA cycle was long considered incomplete because it lacked α-ketoglutarate dehydrogenase, and a modified version of TCA was only discovered a few years ago [56]. Even more recently, a functional Entner–Doudoroff (ED) pathway and a glyoxylate shunt in cyanobacteria have been reported [57]. More importantly, very little quantitative information regarding these central pathways or their regulations has been published. More in-depth investigations and the accumulation of data pertaining to these issues from experiments in systems biology will facilitate the understanding and evaluation of current and future applied engineering strategies. With this information, directions and guidelines for further metabolic engineering efforts can be provided to channel the fixed carbon to creating the desired products. In addition, development of systems biology and metabolic modeling will find more potential engineering targets and guide to metabolic pathway and photosynthesis engineering to further increase chemicals production [58–62].

### Expanding the cyanobacterial genetic toolbox

The development of a genetic toolbox still lags behind what is required for the effective systematic metabolic engineering of cyanobacteria. Although, as mentioned above, a number of constitutive [48, 50] and inducible promoters [35] have been developed or used in cyanobacteria, there is still a need for developing strong, tightly-regulated promoters to augment production pathways that can also be tuned. In addition, because many promoters still work in a generic way, the development of specific, modular promoters is also necessary. Furthermore, the transformation efficiency remains relatively low, and scarless gene disruption methods are required to allow the engineering of multiple genes simultaneously.

### Introducing new carbon-fixation pathways

To increase the carbon-fixation efficiency of the Calvin cycle, the photorespiration effect has been circumvented mainly through two strategies in cyanobacteria: engineering Rubisco to reduce its oxygenase activity [23, 63, 64] and introducing bypass pathways to recycle the photorespiration metabolite 2-phosphoglycolate [33]. However, both efforts showed limited success, perhaps because the CCM of cyanobacteria is already effective at curbing photorespiration. To further improve CO_2_ fixation, a more ambitious and promising strategy is to introduce new pathways, other than the Calvin cycle, into the cyanobacterial cells. Of the six naturally occurring carbon-fixation pathways, the Calvin cycle seems to be the most costly in terms of energy expense [65, 66]. Furthermore, computer simulations suggest that combining natural pathways can create hybrid pathways [67]. New pathway design could also take into account the energy and cofactor requirements for a specific chemical. In this way, carbon fixation and other chemical conversion routes could be efficiently coupled.

### Improving energy supply

In practice, the supply of light energy poses a serious challenge for cyanofactories because of the self-shading effect of cyanobacterial cells. The rapid decrease in sunlight intensity in water also renders a large portion of cells below water surface short of light energy [68]. Besides designing novel bioreactors (this part is not the main focus of this review and thereby not discussed here, see reviews by Chen [69] and Gupta [70]), efforts have been made in synthetic biology in recent years to address this problem.

One strategy is to truncate the antenna system, decreasing the cell’s pigment content (for example, of chlorophyll or phycobilisome) and thereby increasing light penetration [71, 72]. Unfortunately, several studies focusing on antenna truncation have reported a decrease in both growth rate and biomass accumulation [73]. The challenge could also be tackled by providing auxiliary chemical energy. It has been reported that after introducing a bidirectional hydrogenase from *Clostridium*, hydrogen could be used as an energy source to provide NADPH and thereby maintain the viability of *S.* 6803 [74]. In the same vein, other chemical energy sources could also be explored.

### In vivo reconstruction of photosynthetic apparatus

Current understanding of the photosynthetic apparatus is less advanced than that of the cyanobacterial metabolism. However, in recent years considerable achievements have been made to understand the structure, function, and assembly of the photosystem complexes (PSI and PSII) [75, 76]. While the insights gleaned from recent progress have led to success in the conceptual demonstration of mimicking the natural photosystem complexes in vitro [77], there is still a long way to go for in vivo reconstruction of a functional PSI or PSII in a non-photosynthetic host like *E. coli* or yeast. To this end, significant progress is still needed to understand the assembly and regulatory factors of the photosystem complexes.

## Conclusion

In the past decade, significant achievements have been made with the aim of turning cyanobacteria into efficient microbial cell factories, and a few systematically conducted cases have been demonstrated for chemicals such as ethanol, sucrose, isobutanol, lactate and 2,3-butanediol. Yet, cyanobacterial synthesis of many chemicals is still described in a proof-of-concept fashion. Cyanofactories are still far from being efficient, compared with heterotrophic cell factories. Challenges like low photosynthetic efficiency and carbon partitioning towards target chemicals limit the use of cyanobacteria on an industrial scale, and new strategies are needed to address these challenges. Better solutions probably lie in cross-disciplinary efforts, with combined efforts of both cyanobacterial physiologist and metabolic engineers. In the next decades, with the rapid development of systems biology, structural biology and synthetic biology, we can anticipate the generation of much more efficient cyanofactories in terms of photosynthetic and chemical production efficiencies.

## Abbreviations

DCW: dry cell weight
Rubisco: ribulose-1,5-bisphosphate carboxylase/oxygenase
*S*. 7942: *Synechococcus elongatus* PCC 7942
*S*. 6803: *Synechocystis* sp. PCC 6803
RuBP: ribulose-1,5-biphosphate
PRK: phosphoribulokinase
*E. coli*: *Escherichia coli*
*S*. 7002: *Synechococcus* sp. PCC7002
CCM: carbon concentrating mechanism
CA: carbonic anhydrase
PHB: poly-3-hydroxybutyrate
TCA: tricarboxylic acid
LDH: l-lactate dehydrogenase
YqhD: alcohol dehydrogenase
sADH: secondary alcohol dehydrogenase
*S. cerevisiae*: *Saccharomyces cerevisiae*
ED: Entner–Doudoroff
